# The Effects of Combining Aerobic and Heavy Resistance Training on Body Composition, Muscle Hypertrophy, and Exercise Satisfaction in Physically Active Adults

**DOI:** 10.3390/healthcare11172443

**Published:** 2023-08-31

**Authors:** Jerrican Tan, Oleksandr Krasilshchikov, Garry Kuan, Hairul Anuar Hashim, Monira I. Aldhahi, Sameer Badri Al-Mhanna, Georgian Badicu

**Affiliations:** 1 Fitness Innovations Malaysia Sendirian Berhad, Petaling Jaya 47820, Selangor, Malaysia; jerrican@student.usm.my; 2 School of Health Sciences, Universiti Sains Malaysia, Kubang Kerian 16150, Kelantan, Malaysia; garry@usm.my (G.K.); hairulkb@usm.my (H.A.H.); 3 Faculty of Sports Science and Recreation, Universiti Teknologi MARA, Shah Alam 40450, Selangor, Malaysia; 4 Department of Rehabilitation Sciences, College of Health and Rehabilitation Sciences, Princess Nourah bint Abdulrahman University, P.O. Box 84428, Riyadh 11671, Saudi Arabia; mialdhahi@pnu.edu.sa; 5 Department of Physiology, School of Medical Sciences, Universiti Sains Malaysia, Kubang Kerian 16150, Kelantan, Malaysia; sameerbadri9@gmail.com; 6 Department of Physical Education and Special Motricity, Faculty of Physical Education and Mountain Sports, Transilvania University of Braşov, 500068 Braşov, Romania; georgian.badicu@unitbv.ro

**Keywords:** heavy resistance training, aerobic training, body composition, muscle hypertrophy, exercise satisfaction, physically active adults

## Abstract

This study investigated the effects of combined aerobic and heavy resistance training on the variables of body composition, muscle hypertrophy, and exercise satisfaction in physically active adults in comparison with heavy resistance training only (predominantly designed for hypertrophy). Twenty-two healthy male adults between the ages of 18 and 35, who had limited previous experience with muscle resistance training, participated in the intervention program while maintaining their physical activity level. The participants were randomly allocated into two groups: the resistance training group (control group) and the combined training group (experimental group), which involved both resistance training and aerobic training. Aerobic training consisted of 30 min aerobic interval training sessions three times a week with a total of 8 min work bouts in each at 60–70% of heart rate reserve (HRR). The intervention training program lasted for eight weeks. Resistance training consisted of a 3-day muscle group split (2–3 exercises per muscle group, 8 sets per muscle group, 6–12 repetition maximum (RM). Upon completion, body composition, muscle hypertrophy, and exercise satisfaction were analyzed using the mixed-design ANOVA. Variables selected for this study as markers of body composition responded differently to the different interventions and time; however, some trends were not statistically significant. Overall, it is not possible to state unequivocally that one training modality was superior to another in the body composition cluster, for significant improvements were observed within the groups from pre- to post-interventions, but no significant differences were observed between the resistance training and combined training groups, while, both interventions showed improvement with time in some variables of muscle hypertrophy. Compared to baseline, the exercise satisfaction post-intervention improved within the groups. From pre- to post-testing, both resistance and combined training groups improved exercise satisfaction (*p* < 0.05 in both groups). However, there was no significant difference in exercise satisfaction observed between the resistance training and combined training groups after the training intervention (*p* > 0.05).

## 1. Introduction

Muscle hypertrophy results in an increase in the human metabolism rate [1]. The metabolic rate of muscle is estimated to be around 10 to 15 kcal/kg per day, which is equivalent to approximately 4.5 to 7.0 kcal/lbs per day [2]. Studies of physical activity and strength training interventions (lasting from 8 to 52 weeks) showed an increase in muscle mass of about 2.2 to 4.5 lbs [3,4]. This indicates that the increase of 4.5 pounds of muscle mass would increase the resting metabolic rate by about 50 kilocalories per day. Therefore, greater muscle mass results in a higher energy demand by muscle tissue during physical activity. Additionally, participating in aerobic endurance training leads to increased caloric expenditure, which aids in the reduction of body fat [5]. The incorporation of aerobic endurance training within a weight management plan has been identified as a significant factor in achieving optimal health-related outcomes [6,7]. 

Performing aerobic and strength training concurrently is an essential part of physical training aimed at improving not only health but athletic performance as well [8]. Beyond the health-related benefits of combining training modalities in one intervention program, researchers also addressed the fitness-related effects of such combinations. Among the attempts to increase strength component in view of fitness-related outcomes, a study on improving power by combining two strength training modalities within one intervention program proved the efficiency of complex training and its superiority over the traditional resistance training in Malaysia amateur weightlifters with at least 2 years of competitive weightlifting experience at the state level [9].

Aerobic endurance exercise is generally thought to have a limited effect on muscle hypertrophy. This is supported by research suggesting that aerobic exercise activates catabolic pathways, while anaerobic exercise stimulates anabolic pathways [10]. There are differences in the intracellular signaling response between the two types of exercises [11,12]. The findings led to the hypothesis of the AMP-activated protein kinase pathway (AMPK)—phosphatidylinositol 3-kinase (AKT) switch, suggesting that there is a discrepancy in the signaling responses produced by anaerobic and aerobic exercises, which may not complement each other to optimize muscular adaptations. AMPK signaling is for the catabolic pathway and is often associated with aerobic endurance exercises, while AKT signaling is for the anabolic pathway and is often associated with anaerobic or resistance training exercises [12]. However, this theory can be overly simplified and leads to misinterpretation. Multiple studies have shown increased mTOR (mammalian target of rapamycin) activation following aerobic endurance exercise [13]. At the same time, resistance training has consistently been found to increase the levels of AMPK [12]. Both aerobic exercise and resistance exercise have been found to be beneficial for increasing muscle mass, strength, and function. The question remains whether combining these two types of exercise yields superior results compared to performing either exercise modality alone. 

Some recent studies [14,15] indicated no interference effect of aerobic training when combined with strength training. One study [14] concluded that concurrent training, regardless of the exercise order, can be a viable strategy to improve lower-body maximal strength and total lean mass comparably to resistance-only training with no reference to muscle hypertrophy. The same study suggests that conventional resistance training may facilitate increasing strength in concurrent training. No hypertrophy variables were involved in this study either. 

There is limited research on the effects of combining aerobic and resistance exercise on muscle mass in adults. In one study [16], after 5 weeks of intervention, both the strength and strength plus endurance training groups experienced a significantly greater increase in strength (i.e., bench press, biceps curl) and arm cross-sectional area (i.e., the left and right arms) in comparison to the control (no exercise) group. However, there were no significant differences between the strength training group and the strength plus endurance training group. In a small cohort of untrained young males, strength plus endurance training did not impede strength gains or muscle hypertrophy when compared to strength training alone. 

Understanding the effects of combined aerobic and resistance exercise on muscle mass could have important implications for developing beneficial programs that target the individual’s optimal physiological benefit and satisfaction as well. Therefore, the aim of the current study is to investigate the effect of combined aerobic exercise and resistance exercise on muscle-mass-related variables in adults compared to performing resistance training alone. The objective of the study will be achieved through the following specific aims: to assess the effects of combined aerobic and heavy resistance training on body composition and muscle hypertrophy in physically active adults. In addition, the study will also assess the effect of these training regimens on exercise satisfaction in physically active adults.

## 2. Methods

### 2.1. Study Design and Participants 

An experimental study design was conducted to investigate the effect of combining traditional muscle hypertrophy training with aerobic training on muscle mass and other related variables. The study was conducted in a workout studio in Kuala Lumpur, Malaysia, and the research protocol was approved by the Universiti Sains Malaysia Human Ethics Committee Protocol No. USM/JEPeM/19090542.

In this study, a convenience sampling method was applied, and the participants were randomly assigned to two groups: a control group and an experimental group. The control group performed traditional muscle hypertrophy training, which included exercises that targeted specific muscle groups using heavy weights and low repetitions. The experimental group combined traditional muscle hypertrophy training with aerobic training. Both groups underwent an 8-week training program, which consisted of three training sessions per week, with each session lasting approximately one hour. The subjects were monitored by trained instructors to ensure proper form and technique during each exercise session.

The research inclusion criteria in this study were healthy physically active male adults between the ages of 18 and 35. The age range was chosen to avoid hormonal factors that could affect hypertrophy, which typically begins to decline after age 35. Additionally, the study focused exclusively on male participants to reduce any potential confounding effects of gender differences in muscle mass and strength.

To ensure that subjects respond homogeneously to hypertrophy training, WHO general population BMI classification was followed during the subjects’ recruitment; hence the participants were expected to have a BMI of between 18.5 and 24.9 kg/m^2^.

The study included participants who were familiar with resistance training and previously practiced free-weight exercises but had not practiced muscle-hypertrophy-oriented resistance training before. Their history of involvement was movement and health oriented. Their previous training volumes (sets per muscle group) were low, not exceeding 1–2 sets per muscle group per week. This was done to ensure that participants had not previously developed substantial muscle mass or strength, which could confound the results of the study. However, the study did include physically active adults who were regularly engaged in health-related fitness activities and exercised at least 3 times per week for an average of 60 min over the past 6 months. Any participants reported to have sedentary behavior, or a history of cardiovascular disease were excluded from the study.

### 2.2. Sample Size Calculation 

The sample size for this study was estimated using a statistical power analysis. The strength was set at 0.80 and the confidence level was set at 95%, which is a common level of significance in research studies. Based on these parameters, the sample size was calculated to be 20 participants. However, to account for possible dropouts during the intervention period (expected to not exceed 20%), 24 subjects were recruited for the study with each study group containing 12 participants. 

### 2.3. Study Outcomes 

Research variables’ clusters in the study were body composition, muscle hypertrophy, and exercise satisfaction, which were measured as follows:

Body composition: Data were collected using a Bioimpedance Analysis Machine (Tanita, Japan). Percentage of fat mass, body mass index, fat mass (kg), and lean body mass (kg) were calculated. All the procedures were followed by the trained fitness practitioner as prescribed by the equipment manufacturer’s instructions. 

Muscle hypertrophy: Upper and lower limb girths, chest girth (mm), shoulder girth (mm), waist girth (mm), thigh girth (mm), and hip girth (mm) were measured. To ensure accuracy, standards from the International Society for the Advancement of Kinanthropometry (ISAK) were followed. All the measurements were conducted with body and limbs relaxed, with no residual effects from the previous/last resistance training session.

Exercise satisfaction scale: An 8-item version of the Physical Activity Enjoyment Scale (PACES) from [17] that provided a valid instrument for assessing enjoyment in physical activity was distributed among the participants. The questionnaire was duly validated for Malaysian participants [18]. 

Respondents were asked to rate “how you feel at the moment about the physical activity you have been doing” using a 7-point Likert scale (1 = un-pleasurable; 7 = pleasurable). Two items were reverse-coded. The sum of all the items forms a unidimensional measure of enjoyment. Higher values reflect greater levels of enjoyment.

### 2.4. Training Intervention

Intervention protocols included classic hypertrophy-oriented resistance training alone and a combination of hypertrophy-oriented resistance training with aerobic training. Both groups underwent orientation (familiarization) to learn exercise techniques, familiarize themselves with exercise intensity, and set realistic training expectations. A briefing on suggested meals was also arranged. In the control group, the training protocol included 3 sessions per week, 45–60 min per session (Table 1, Table 2 and Table 3).

Training exercises included the use of barbells and dumbbells. To ensure maximal safety for the participants, particularly those without previous hypertrophy/heavy weights experience, 1 RM testing and subsequent conversions were replaced by the practical weight selection approach, whereby to determine the load to be lifted for 10 RM, we were determined the load at which the participant could do 10 RM. Instructors involved in the study observed and ensured that the last repetition was coming 1–2 reps before muscle fatigue/failure. 

Progression (when necessary) was ensured through the application of the same practical approach on the weights lifted, through the adjustments of the weight to ensure that the same RM is maintained post-progression.

Experimental group (Resistance + Aerobic) training intervention protocol included 3 sessions of resistance training per week plus 3 sessions of aerobic training of 30 min total duration (Table 4).

This protocol was used in the experimental training group only. Resistance training was the same as with control group sessions.

As the above shows, the research protocol used in the training intervention for the combined training (experimental) group was 90 min per week longer in duration than the intervention protocol for the resistance (control) training group.

Counting the work bouts per session, however (with each work bout per aerobic training session being 8 min), the workload difference between sessions in the two protocols was 24 min per week. The rest of the time was spent in warm-up, cool-down, and rest intervals (all at <60% of HRR).

### 2.5. Data Analysis

The normality distribution of the study parameter scores was examined using the histogram plot and the Kolmogorov–Smirnov test for inferential statistics, and the scores were found to be normally distributed (*p* > 0.05), requiring the use of a parametric test. The data are presented as mean and standard deviation for continuous variables and frequency and percentage for categorical variables. The mixed-design ANOVA was performed to determine the mean differences of the study parameters between group effects (control and intervention groups), within-group effects (across time), and the interaction effect (group × time). All statistical analyses were performed using the Statistical Product and Service Solution (SPSS) version 27. The level of statistical significance was set at a *p*-value of <0.05.

## 3. Results

**Figure 1:**
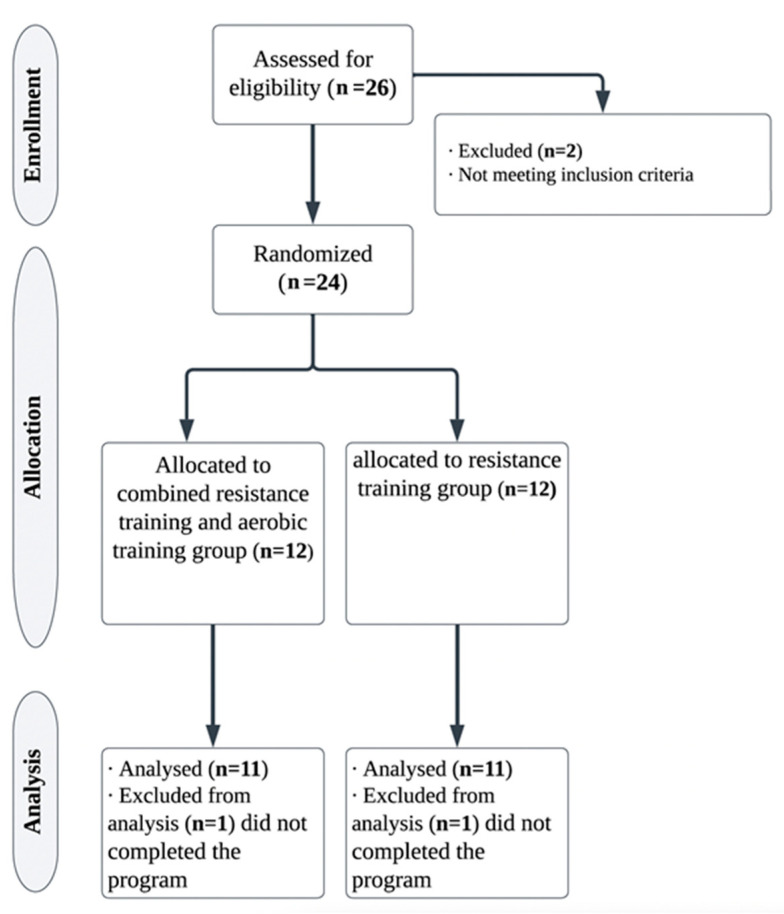
CONSORT flowchart of the study.

A total of 24 participants took part in the study, 12 who were to receive resistance hypertrophy-focused training only, and 12 who were to receive combined resistance and aerobic training. Out of the original 24 participants recruited to the study, one participant from each group discontinued (due to the non-adherence to the intervention program) their participation in the intervention program, hence 22 (11 participants per group) were eventually analyzed statistically (Figure 1).

The participants had a mean age and mean height of 26.68 (SD = 4.34) and 172.00 (SD = 5.94), respectively.

### 3.1. Effects of Resistance Training & Combined Training on Body Composition

Analyzing the within-group effect, there were no significant differences observed in the resistance training group, whereas the combined group exhibited significant changes in some variables. 

Weight loss was observed from the mid- to post-test (*p* = 0.006) period, signifying those participants lost weight due to the intervention training (Table 5). 

Within-group changes were observed in body fat percentage, including a significant (*p* = 0.003) and (*p* = 0.016) reduction in the fat percentage from pre- to mid-intervention and pre- to post-intervention, respectively, in the combined training group (Table 5). 

The dynamics of the fat mass data included within-group improvements in the fat mass, namely the significant (*p* = 0.007) and (*p* = 0.021) reduction in the fat mass from pre- to mid-intervention and pre- to post-intervention, respectively, in the combined training group (Table 5) with no significant changes observed in the resistance training group.

Lean body mass fitted into a similar dynamic with significant improvements observed from pre- to mid-intervention (*p* = 0.001) and from pre- to post-intervention (*p* = 0.015) assessments (Table 5). 

No significant interactions between groups were observed in the body composition variables at any measurement point in this study.

### 3.2. Effects of Resistance Training and Combined Training on Muscle Hypertrophy

Among the muscle hypertrophy variables, chest girth improved from pre- to post-intervention testing for resistance and combined training (*p* = 0.029 and *p* = 0.004, respectively) (Table 6). 

Shoulder girth and hip girth improved significantly only in the resistance training group (*p* = 0.002 and *p* = 0.009 respectively) with no improvements in the combined group (Table 6).

Thigh girth, on the contrary, improved in the combined training group (*p* = 0.004) with no improvement of this variable observed in the resistance training group (Table 6). 

No significant interactions between groups were observed in the muscle hypertrophy variables at any measurement point in this study.

### 3.3. Effects of Resistance Training and Combined Training on Exercise Satisfaction

Exercise satisfaction improved from the pre-test to post-test periods within the groups. Both resistance training and combined training groups showed improved satisfaction from pre- to post-testing (Table 7). There was, however, no significant difference observed between the groups (resistance training versus combined training) before the intervention and after training.

## 4. Discussion

Among the body composition variables, significant weight reductions observed in the combined group could be possibly related to the higher caloric expenditures during training with the said group, which were facilitated by the additional aerobic training component. The first 4 weeks of training possibly served as a cumulation time for the adaptations, which materialized and became noticeable later, between weeks 4 and 8. Previous research has demonstrated that a combination of aerobic and resistance exercise is quite beneficial in helping individuals lose weight [19,20,21].

At the same time, neither of the training programs proved more beneficial than the other in body weight loss/gain. It is possible that 8 weeks of training was not long enough to widen the observed changes and to produce significant differences between the groups in terms of body weight. Had the intervention period been longer, such differences could have become significant. The compliance, however, could be compromised by longer intervention and may have led to more participants discontinuing training toward the completion of the intervention. Previous studies used a longer intervention duration than 12 weeks [21], yielding mixed results that were significant in some studies [22] but not in others [23]. In the present study, we were targeting the best balance between the optimal intervention duration and the best possible training program adherence. So optimal outcomes could be achieved with minimum possible training program non-compliance.

Comparing the effects of resistance training and combined training on body fat, the latter brought some noticeable improvements; however, the difference between groups did not reach the desired level of significance. Earlier studies found that the combined group lost more body fat than the resistance training group [21,24,25].

In the current study, body fat improved more in the first 4 weeks of the intervention in the combined training group, whereas body weight reduced significantly in the second part of the intervention in the combined training group. Regardless, by linking these two variables, it becomes obvious that the observed significant weight reduction in the combined training group was due to significant reduction in body fat.

There was a significant mean difference in fat mass with time (*p* = 0.044). Some statistically significant improvements were observed within the combined group from pre- to mid-testing (*p* = 0.007) and from pre- to post-testing (*p* = 0.021).

There was, however, no significant mean difference in fat mass between the resistance training group and the combined training group. However, compared to past studies [21,26,27], the results reveal that the combined group reduced more body fat than the resistance training group.

Lean body mass improved from the pre-test to mid-test (*p* = 0.001) periods and from the pre-test to post-test (*p* = 0.015) periods within the combined training group. Resistance training alone did not facilitate any improvements from pre-training to mid-training and from mid-training to post-training; however, some close to statistical significance levels of lean body mass were observed from pre- to mid-tests in the resistance training group. Hence, adding aerobic components to resistance training significantly influenced the improvement in lean body mass, which is in line with earlier studies [28].

Among the muscle hypertrophy variables, there were mixed effects and improvements for both the resistance training and combined training groups. Namely, shoulder and hip girth were improved by resistance training, whereas thigh girth improved in the combined group only and chest girth improved as the result of training in both groups. 

The observed improvements in chest girth can be attributed to various mechanisms. Some improvements can be related to the possible chest expansion facilitated using exercises like barbell pullovers, wide grip bench presses, and others in resistance training programs [29]. In our study, however, we cannot detect this mechanism since no increase in pectoralis mass was seen as such. The second mechanism can be related to the increased volumes of aerobic training in the combined group, leading to a possible increase in chest expansion [30,31]. That too, however, cannot be confirmed within the framework of the current study and using current research measurement instruments.

Resistance training resulted in increased shoulder girth, demonstrating the benefits of such a training modality. Traditional resistance training may be more efficient if the specific objective is to improve muscle hypertrophy as opposed to more functional kinds of exercise that just result in increases in shoulder girth [32,33].

In common with past research [21,34,35], the findings show that combined training increases thigh girth more than resistance training.

In terms of hip girth, the resistance training group showed a greater improvement, and this training modality looks more effective than combined training for this variable (although statistically insignificant). As mentioned earlier, a previous study reported that combining the two different training strategies may compromise muscle hypertrophy [34].

In the exercise satisfaction analysis, there was no significant mean difference in exercise satisfaction between the resistance training group and the combined training group. Similarly, although the means of exercise satisfaction were slightly higher at the end of the training intervention in the combined training group, the differences were not statistically significant.

Exercise satisfaction improving because of various training modalities is well documented [36,37]. The results of this study confirm an increase in exercise satisfaction in the resistance training group, indirectly suggesting that the training program was enjoyable as such and that training outcomes were achieved. However, adding the aerobic component to the resistance training didn’t alter the attitude to training and satisfaction of the exercisers. The program remained satisfying and enjoyable, with training objectives being achieved as well.

### Limitations of the Study

Although the study was carefully designed and executed, it is important to acknowledge some limitations that could affect the generalizability of the study results. One such limitation of the study is the relatively small sample size. Although the sample size was carefully calculated using a statistical power analysis, a larger sample size could have increased the statistical power of the study and reduced the possibility of type II errors. Additionally, a larger sample size may have increased the generalizability of the study results to other populations.

Another limitation of the study is that objective methods to measure muscle mass directly were not used to assess muscle hypertrophy. Instead, the study used indirect measures of muscle mass, such as circumferences, which are prone to measurement error. Although the study used standardized techniques for taking measurements, objective measures such as MRI or DEXA scans could have possibly provided more accurate and reliable measurements of muscle hypertrophy.

Despite these limitations, the study provides valuable insights into the effects of combined aerobic and resistance exercise on body composition, muscle hypertrophy, and exercise satisfaction.

## 5. Conclusions

Combining heavy resistance training with aerobic training positively influenced the variables of body composition in physically active males, leading to reduced body weight, body fat, and fat mass, and increased lean body mass. 

Adding aerobic components to muscle-hypertrophy-focused training impacted the hypertrophy variables too. It led to thigh girth and chest girth improvements in the combined training group, proving that aerobic training does not oppose the effects of hypertrophy training and instead can facilitate these if resistance and aerobic training are combined in the right proportions. 

The combination of resistance and aerobic training improves exercise satisfaction, as does resistance training alone. Hence, such a combination may be useful in solidifying exercise adherence in prolonged exercise interventions, making them more variable and satisfying.

According to the within-group effects analysis, there were no significant changes elicited by the resistance training in the control group in any of the body composition variables assessed in this study. Therefore, eight weeks of resistance hypertrophy-focused training were not sufficient to enforce the significant changes in the body composition domain. Among the muscle hypertrophy markers, resistance training resulted in significant improvements in chest, shoulder, and hip girths. Resistance training also improved the control group’s level of exercise satisfaction.

## Figures and Tables

**Table 1:** Session/Day 1. 45–60 min per session. Used in both experimental and control groups.

Exercise	Reps	Rest Interval	Sets
Squats	10–12 RM	60 s	4
Romanian Deadlifts	10–12 RM	60 s	4
Leg Press/Leg Extension	10–12 RM	60 s	4

**Table 2:** Session/Day 2. 45–60 min per session. Used in both experimental and control groups.

Exercise	Reps	Rest Interval	Sets
Bench Press	10–12 RM	60 s	4
Incline Chest Press	10–12 RM	60 s	4
Shoulder Press	10–12 RM	60 s	4
Upright Row	10–12 RM	60 s	4
Lying Triceps Extension	10–12 RM	60 s	3

**Table 3:** Session/Day 3 45–60 min per session. Used in both experimental and control groups.

Exercise	Reps	Rest Interval	Sets
Bent-over Row	10–12 RM	60 s	4
Lat Pull-down	10–12 RM	60 s	4
Seated Low Row	10–12 RM	60 s	4
Biceps Curl	10–12 RM	60 s	3

**Table 4:** Interval Aerobic Training. 30–35 min total duration per session three times a week.

	Work Intensity	Work Duration	Recovery Intensity	Recovery Duration	Rounds
Warm-up	Up to 60% HRR	5 min			
Jog	60–70% HRR	2 min	50–60% HRR	4 min	4
Cool-down		5 min			

**Table 5:** Resistance Group and Combined Group within-group effect in body composition variables (Time effect).

Variable	Pre-Intervention		*p*- Value	Mid-Intervention		*p*- Value	Post-Intervention		*p*- Value
	Mean ± SD	MD (95% CI)		Mean ± SD	MD (95% CI)		Mean ± SD	MD (95% CI)	
Body Weight (kg)									
Resistance	71.6 ± 12.2	−0.42 (−1.49, 0.65)	0.42	72.1 ± 13.3	0.17 (−0.60, 0.94)	0.64	71.9 ± 13.2	−0.25 (−1.85, 1.35)	0.75
Combined	83.1 ± 13.2	0.1 (−1.1, 1.4)	0.8	82.9 ± 12.8	1.3 (0.4, 2.2)	0.006 *	81.6 ± 11.6	1.5 (−0.3, 3.3)	0.1
Body Fat (%)									
Resistance	21.5 ± 5.5	0.62 (−0.38, 1.61)	0.2	20.9 ± 5.8	0.12 (−0.93, 1.17)	0.81	20.7 ± 5.99	0.74 (−0.92, 2.39)	0.36
Combined	23.8 ± 8.8	1.8 (0.7, 3.0)	0.003 *	21.9 ± 9.1	0.5 (−0.6, 1.8)	0.32	21.3 ± 7.7	2.46 (0.5, 4.4)	0.016 *
Fat Mass (kg)									
Resistance	15.8 ± 6.2	0.20 (−0.76, 1.16)	0.67	15.6 ± 6.99	0.09 (−1.02, 1.21)	0.86	15.4 ± 7.4	0.29 (−1.54, 2.12)	0.74
Combined	19.3 ± 8.9	1.6 (0.5, 2.7)	0.007 *	19.0 ± 9.7	0.9 (−0.3, 2.2)	0.14	18.1 ± 8.1	2.59 (0.4, 4.7)	0.021 *
Lean Body Mass (kg)									
Resistance	55.8 ± 7.2	−0.63 (−1.29, 0.04)	0.06	56.4 ± 6.98	0.09 (−0.51, 0.70)	0.75	56.3 ± 6.6	−0.54 (−1.25, 0.17)	0.13
Combined	62.4 ± 6.1	−1.4 (−2.2, −0.6)	0.001 *	63.9 ± 6.2	0.3 (−0.3, 1.1)	0.26	63.5 ± 5.7	−1.1 (−1.9, −0.2)	0.01 *

* Denotes significant differences. MD: Mean deviation; SD: Standard deviation, CI: Confidence interval

**Table 6:** Resistance Group and Combined Group within-group effect in muscle hypertrophy variables (Time effect).

Variable	Group	Pre-Intervention		Post-Intervention		*p*-Values
		Mean ± SD	95% CI	Mean ± SD	95% CI	
Chest Girth (cm)	Resistance	96.18 ± 8.10	91.70, 100.66	98.00 ± 8.60	93.14, 102.87	0.029 *
	Combined	101.73 ± 5.98	97.25, 106.21	104.00 ± 6.77	99.36, 109.09	0.004 *
Shoulder Girth (cm)	Resistance	112.91 ± 7.97	108.41, 117.41	115.82 ± 7.72	110.81, 120.83	0.002 *
	Combined	121.09 ± 6.23	116.59, 125.59	122.00 ± 8.21	116.99, 127.01	0.284
Waist Girth (cm)	Resistance	81.14 ± 8.00	76.08, 86.19	82.55 ± 8.04	77.80, 87.29	0.122
	Combined	88.64 ± 8.06	83.58, 93.69	88.14 ± 7.01	83.39, 92.88	0.573
Thigh Girth (cm)	Resistance	60.96 ± 9.79	55.97, 65.94	61.36 ± 9.54	56.38, 66.35	0.477
	Combined	62.64 ± 5.48	57.65, 67.63	64.46 ± 5.87	59.47, 69.44	0.004 *
Hip Girth (cm)	Resistance	97.18 ± 8.71	92.17, 102.19	98.64 ± 8.97	93.52, 103.75	0.009 *
	Combined	103.82 ± 7.14	98.81, 108.83	103.46 ± 7.20	98.34, 108.57	0.477

* *p* ≤ 0.05.

**Table 7:** Resistance Group and Combined Group within-group effect in exercise satisfaction (Time effect).

Variable	Group	Pre-Intervention	Post-Intervention	*p*-Values
		Mean ± SD (95% CI)	Mean ± SD (95% CI)	
Exercise satisfaction	Resistance	37.46 ± 9.94 (31.94, 42.97)	48.91 ± 6.43 (45.38, 52.44)	<0.001 *
	Combined	37.36 ± 7.40 (31.85, 42.88)	50.36 ± 4.65 (46.84, 53.89)	<0.001 *

* *p* ≤ 0.05.

## Data Availability

Data will be available on request.
